# Transcriptome Changes in *Pseudomonas putida* KT2440 during Medium-Chain-Length Polyhydroxyalkanoate Synthesis Induced by Nitrogen Limitation

**DOI:** 10.3390/ijms22010152

**Published:** 2020-12-25

**Authors:** Dorota Dabrowska, Justyna Mozejko-Ciesielska, Tomasz Pokój, Slawomir Ciesielski

**Affiliations:** 1 Department of Environmental Biotechnology, University of Warmia and Mazury in Olsztyn, 10-719 Olsztyn, Poland; dorota.dabrowska@uwm.edu.pl (D.D.); tomasz.pokoj@uwm.edu.pl (T.P.); 2 Department of Microbiology and Mycology, University of Warmia and Mazury in Olsztyn, 10-719 Olsztyn, Poland; justyna.mozejko@uwm.edu.pl

**Keywords:** biopolymers, gene expression, mcl-PHAs, *Pseudomonas putida* KT2440, RNA-seq, transcriptional regulators, transcriptomics

## Abstract

*Pseudomonas putida*’s versatility and metabolic flexibility make it an ideal biotechnological platform for producing valuable chemicals, such as medium-chain-length polyhydroxyalkanoates (mcl-PHAs), which are considered the next generation bioplastics. This bacterium responds to environmental stimuli by rearranging its metabolism to improve its fitness and increase its chances of survival in harsh environments. Mcl-PHAs play an important role in central metabolism, serving as a reservoir of carbon and energy. Due to the complexity of mcl-PHAs’ metabolism, the manner in which *P. putida* changes its transcriptome to favor mcl-PHA synthesis in response to environmental stimuli remains unclear. Therefore, our objective was to investigate how the *P. putida* KT2440 wild type and mutants adjust their transcriptomes to synthesize mcl-PHAs in response to nitrogen limitation when supplied with sodium gluconate as an external carbon source. We found that, under nitrogen limitation, mcl-PHA accumulation is significantly lower in the mutant deficient in the stringent response than in the wild type or the *rpoN* mutant. Transcriptome analysis revealed that, under N-limiting conditions, 24 genes were downregulated and 21 were upregulated that were common to all three strains. Additionally, potential regulators of these genes were identified: the global anaerobic regulator (Anr, consisting of FnrA, Fnrb, and FnrC), NorR, NasT, the sigma^54^-dependent transcriptional regulator, and the dual component NtrB/NtrC regulator all appear to play important roles in transcriptome rearrangement under N-limiting conditions. The role of these regulators in mcl-PHA synthesis is discussed.

## 1. Introduction

Polyhydroxyalkanaotes (PHAs) have been attracting interest for years due to their unique properties, particularly the fact that they are degraded in soil, water, compost, or marine sediments. In addition, PHAs exhibit optical activity, they have antioxidant properties, they are piezoelectric and biocompatible, and they can be thermally processed [1]. Moreover, functional groups that could enhance their desirable properties can be incorporated into PHAs via chemical modifications [2]. All of these attributes make them promising biomaterials that could replace petrochemical-based plastics in the future.

Polyhydroxyalkanoates (PHAs) are synthesized by microorganisms for energy storage under unbalanced growth conditions; they take the form of granules in the cellular structure [3]. The ability to synthesize and accumulate PHAs is widespread among bacteria, and PHA metabolism influences many cell activities [4]. PHAs have generally been classified according to their monomer size as either short-chain-length PHAs (scl-PHA) with C4–C5 monomers, or medium-chain-length PHAs (mcl-PHAs) with C6–C14 monomers [5]. Whereas scl-PHAs can be synthesized by numerous Gram-positive and Gram-negative bacteria, mcl-PHAs are synthesized mainly by bacteria of *Pseudomonas* species [6]. These two types of PHAs differ tremendously in their thermal properties and mechanical behavior [7]. While scl-PHAs are brittle and tend to have high crystallinity, mcl-PHAs are more flexible, which makes them more suitable biomaterials, especially for medical applications [7,8,9].

One of the best known mcl-PHA producers is *Pseudomonas putida* KT2440, which due to its reputation as an efficient cell factory, has become a model bacterium in biotechnology [10]. This bacterium encodes a large number of genes related to the utilization of unusual compounds as carbon sources [11]. The large number of proteins that it synthesizes means that gene regulation in this bacterium is a complicated system. The *P. putida* KT2440 genome codes for more than 600 transcriptional factors and more than 24 alternative sigma subunits of RNA polymerase [12]. As a result, the gene regulation system in this species is relatively poorly understood. Recently, however, progress in high-throughput technologies such as transcriptomics, proteomics, and metabolomics has made it possible to understand the relationships between genetic regulation and metabolic activity.

To metabolize and accumulate mcl-PHAs, pseudomonads use different pathways, depending on the type of external carbon source. Most mcl-PHA intermediates are obtained through ß-oxidation of fatty acids if the carbon source is an alkane or fatty acid, whereas non-PHA related carbon sources, such as acetate, ethanol, glycerol, glucose, and gluconate, can be oxidized to acetyl-CoA and channeled towards PHA formation via the de novo fatty acid synthesis pathway [4,13]. In the end, PHAs are synthesized and accumulated by genes organized in two main operons [14]. The main operon, *phaC1ZC2D*, consists of genes coding for two polymerases (PhaC1 and PhaC2), a depolymerase (PhaZ), and a transcriptional activator (PhaD). The second operon, *phaIF*, comprises *phaF* and *phaI*, which code for proteins involved in granule formation, an essential part of accumulating and synthesizing these biopolyesters. PhaD is the transcriptional activator that controls the transcription of both operons [15]. Additionally, there is *phaG*, which is not co-localized with the *pha* gene cluster. This gene encodes transacylase, which is also involved in synthesis of mcl-PHAs from structurally non-related carbon sources [16].

PHAs are accumulated by *Pseudomonas* species under unfavorable growth conditions, particularly when nitrogen, phosphorus, and oxygen are limited [17,18,19]. In spite of many papers dealing with mcl-PHAs synthesis, the molecular mechanisms that cause *Pseudomonas* to respond to nitrogen limitation by synthesizing and accumulating PHAs are still unknown. In particular, there is a lack of information about the transcriptional regulators that drive the processes of mcl-PHA synthesis and accumulation.

To investigate the mechanisms by which nitrogen limitation influences the synthesis of PHAs, mutants deficient in the synthesis of the alternative sigma factor RpoN (sigma^54^) could be helpful. Such a mutant cannot activate genes that are regulated by nitrogen limitation because it cannot synthesize sigma^54^, an alternative subunit of RNA polymerase. Additionally, the *P. putida* KT2440 *rpoN* mutant has been used in work of Hoffman and Rehm (2004), in which the effect of nitrogen limitation on mcl-PHAs synthesis was analyzed. The *P. putida* KT2440 *rpoN* mutant showed significant differences in mcl-PHA concentration between optimal and nitrogen limiting conditions, as did the wild form. However, when octanoate was used as the external carbon source, there was a significant difference between the *rpoN* mutant and the wild form in terms of mcl-PHA concentration, suggesting that mcl-PHA synthesis is affected by RpoN. However, the molecular basis of this observation has not been revealed yet.

It was also shown that there is relationship between PHA synthesis and the stringent response mechanism that modifies the physiology of the bacterium under nutrient limitation and stresses [20,21]. This response is mediated by the alarmons—unusual nucleotides, guanosine tetraphosphate (ppGpp), and guanosine pentaphosphate (pppGpp), often referred to collectively as (p)ppGpp, which primarily affects the transcriptional program of the bacterial cell [22]. Mozejko-Ciesielska et al. (2017) revealed that the *P. putida* KT2440 *relA*/*spoT* mutant synthesized similar amounts of mcl-PHAs in both optimal and nitrogen limiting conditions when oleic acid was used as the external carbon source [21]. As the amount of accumulated PHAs was lower than in cells of wild type *P. putida* KT2440, and additionally, the *relA*/*spoT* mutant showed elevated levels of expression of some PHA-related genes, it was suggested that the stringent response can influence mcl-PHA synthesis. The molecular factors joining the stringent response and mcl-PHA synthesis are still waiting to be uncovered. There is also a lack of information about the possibility, when using other carbon sources, of mcl-PHA synthesis by a mutant deficient in the stringent response. This information could be helpful in understanding which metabolic pathways can be affected by the stringent response.

The first specific goal of this work was to compare the growth and efficiency of mcl-PHA synthesis between *P. putida* KT2440 wild type and their two mutants as a result of growing in optimal and nitrogen limiting conditions. Additionally, we aimed at revealing the differences in the expression of main genes responsible for mcl-PHA synthesis and in the accumulation between analyzed strains dependent on growth conditions. Finally, we examined the global changes of *P. putida* KT2440 wild type and mutant transcriptomes due to nitrogen limitation in relation to mcl-PHA synthesis using sodium gluconate. Overall, the main goal was to examine and indicate genes driving the transcriptomic reaction to nitrogen limitation. 

## 2. Results

### 2.1. PHA Synthesis 

**Figure 1:**
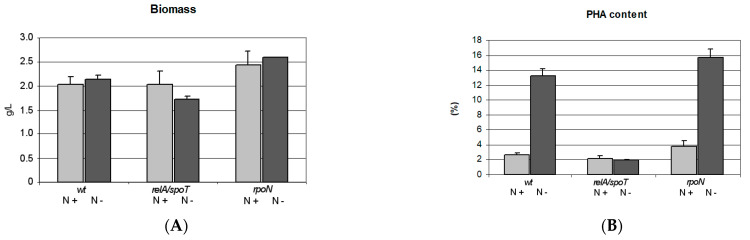
Cell dry mass (**A**) and PHA content (**B**) of wild type of *Pseudomonas putida* KT2440 and its *relA/spoT* and *rpoN* mutants. Shake flask cultivation was performed under optimal (N+) and nitrogen limiting conditions (N−). Mean values with standard deviations are shown (n = 6).

The cultivations of *P. putida* KT2440 wild type and their mutants were performed in shaking flasks using medium supplemented with sodium gluconate as the only carbon source. All cultivations were carried out in six replicates under optimal growth conditions and under nitrogen limitation. After 48 h of growth, final cell dry mass (CDM) ranged from 1.7 ± 0.1 to 2.6 ± 0.0 g/L (Figure 1). The highest biomass was found with the *rpoN* mutant cultivated under nitrogen limiting conditions, whereas the lowest with the *relA*/*spoT* mutant cultivated in this same conditions. The only statistically significant difference (<0.05) in biomass amount was shown for this two samples. There were no differences in biomass due to different conditions between the studied strains. Under nitrogen limitation, the wild-form and *rpoN* mutant accumulated the largest amounts of mcl-PHAs (13.2 ± 0.9% and 15.6 ± 1.2% mcl-PHAs of CDM, respectively). Under optimal conditions, these two strains accumulated significantly less mcl-PHAs (2.6 ± 0.3 and 3.8 ± 0.7% mcl-PHAs of CDM, respectively; *p* < 0.05). The *relA*/*spoT* mutant synthesized similar amounts of mcl-PHAs in both optimal and nitrogen limiting conditions (2.2 ± 0.4 and 1.9 ± 0.1% mcl-PHAs of CDM, respectively). In nitrogen limiting conditions the PHA concentration in *relA*/*spoT* mutant cells was significantly lower than in wild type and *rpoN* mutant cells (<0.05).

Ammonium concentrations were measured at 24 and 48 h of cultivation. In optimal conditions in 24 h of cultivation, the ammonium concentrations were 2.23, 2.08, and 2.03 g NH_4_^+^-N/L in the wild type, *relA*/*spoT* mutant, and *rpoN* mutant, respectively. In nitrogen limiting conditions ammonium was completely consumed in 24 h. At 48 h of cultivation, the ammonium concentrations were 2.1, 1.7, and 1.63 g NH_4_^+^-N/L in the wild type, *relA*/*spoT* mutant, and *rpoN* mutant, respectively.

**Figure 2:**
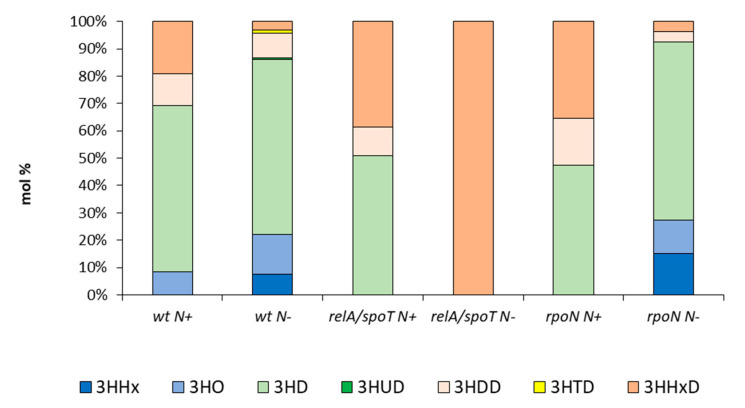
Monomeric composition of mcl-PHAs synthesized by the wild type of *Pseudomonas putida* KT2440 and the *relA/spoT* and *rpoN* mutants grown under optimal (N+) and nitrogen limiting conditions (N−). Abbreviations: 3HHx, 3-hydroxyhexanoate; 3HO, 3-hydroxyoctanoate; 3HD, 3-hydroxydecanoate; 3HUD, 3-hydroxyundecanoate; 3HDD, 3-hydroxydodecanoate; 3HTD, 3-hydroxytetradecanoate; 3HHxD, 3-hydroxyhexadecanoate.

The monomeric composition of purified mcl-PHAs was analyzed using gas chromatography. The major repeat unit of the mcl-PHAs produced by the analyzed strains in both conditions was 3-hydroxydecanoate. The only exception was the mcl-PHA accumulated by the *relA*/*spoT* mutant in nitrogen limiting conditions, which comprised only 3-hydroxyhexadecanoate (3HHxD). With the exception of this mutant, it was shown that nitrogen limitation decreased the molar concentrations of 3-hydroxydodecanoate (3HDD) and 3-hydroxyhexadecanoate (3HHxD, Figure 2).

### 2.2. Analysis of mcl-PHA Related Genes Using Reverse Transcription Real-Time PCR

**Figure 3:**
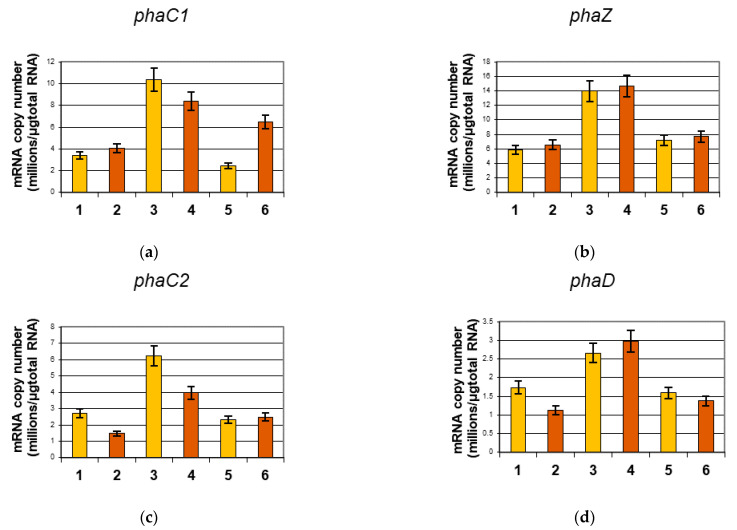

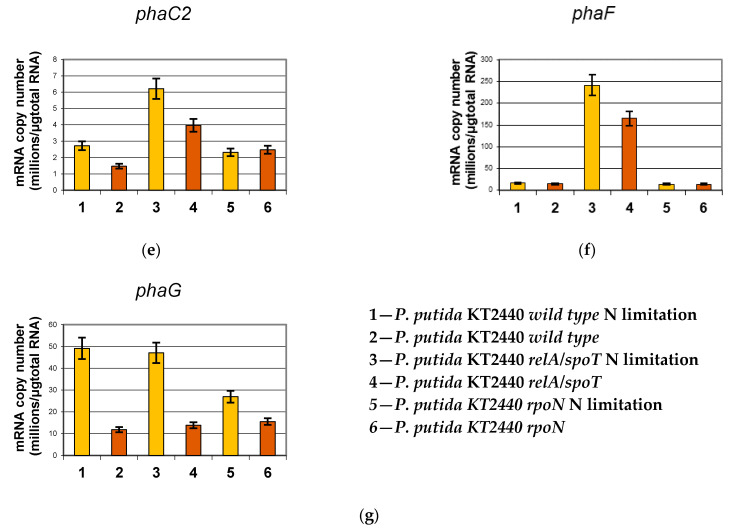
The results of quantitative real-time reverse transcription PCR analysis of *phaC1* (**a**), *phaZ* (**b**), *phaC2* (**c**), *phaD* (**d**), *phaI* (**e**), *phaF (***f***)*, and *phaG (***g***)* gene expression. Samples were taken at 48 h of cultivation. All analyzed strains were cultivated in shake flasks under optimal and nitrogen limiting conditions. Mean values with standard deviations are shown (*n* = 3). 1—*P. putida* KT2440 wild type N limitation; 2—*P. putida* KT2440 wild type; 3—*P. putida* KT2440 *relA*/*spoT* N limitation; 4—*P. putida* KT2440 *relA*/*spoT*; 5—*P. putida* KT2440 *rpoN* N limitation; 6—*P. putida* KT2440 *rpoN.*

The transcriptional expression levels of the *phaC1*, *phaZ*, *phaC2*, *phaD*, *phaI*, *phaF*, and *phaG* genes were examined at 48 h of cultivation. The results in Figure 3 show that the mRNA copy numbers varied between the analyzed strains and conditions. Nitrogen limiting conditions did not increase transcription none of gene from the *phaC1ZC2D* operon in statistically significant manner (*p* < 0.05). The highest expression levels of these genes were observed in the *relA*/*spoT* mutant cells (Figure 3). Generally, in the *phaC1ZC2D* operon the highest expression was noticed in the *phaZ* gene, especially in case of the *relA*/*spoT* mutant. The expression of genes comprising the *phaIF* operon was almost 40 times higher than expression of genes creating the *phaC1ZC2D* operon. The numbers of *phaI* and *phaF* gene transcripts were significantly higher in the *relA*/*spoT* mutant than in wild type and the *rpoN* mutant. Moreover, nitrogen limitation increased the expression of *phaI* and *phaF* genes in this mutant in a statistically significant manner (*p* < 0.05). The expression of the *phaG* gene showed also the influence of nitrogen limitation. In all analyzed strains, expression of the *phaG* gene was elevated in conditions when nitrogen was completely depleted. Generally, except for the *phaG* gene, the *relA*/*spoT* mutant exhibited the highest expression of PHA related genes. Only in the case of *phaG* gene expression was there a lack of any differences between the wild type and the *relA*/*spoT* mutant. The numbers of all analyzed gene transcripts were at comparable levels in the wild type and *rpoN* mutant.

### 2.3. RNA-Seq

A total of about 200-million raw sequencing reads were generated from RNAseq data, with an average of 34 million reads per sample. After the elimination of low-quality reads with multiple N, reads shorter than 20 bp, and removal of sequences coding for rRNA, a total of 114 million qualified mRNA sequence reads were mapped onto the *Pseudomonas putida* KT2440 genome [23]. The obtained sequence reads matched to 5563 coding genes of the *P. putida* KT2440 genome [23]. After expression quantification, differential gene expression analysis was carried out between all analyzed samples. The expression levels for each gene were quantified as reads per kilobase per million mapped reads (RPKM), as described by Mortazavi et al. (2008) [24].

**Figure 4:**
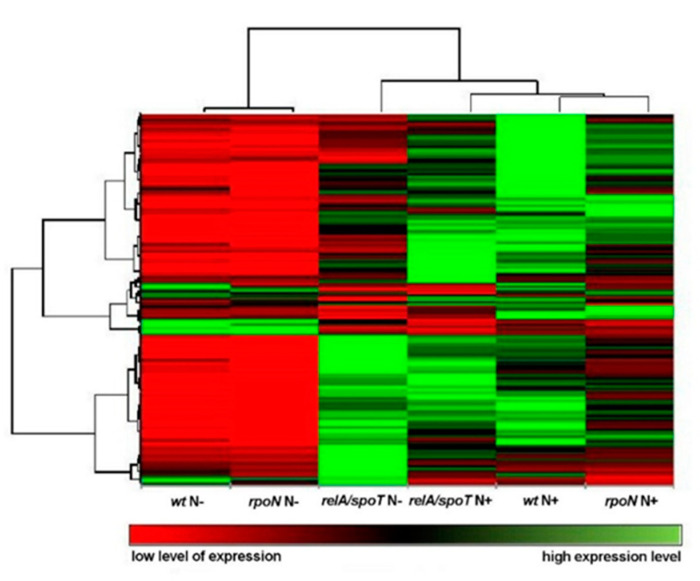
Heatmap of differential gene expression profiles between all analyzed strains, values given in RPKM (reads per kilobase million). *Pseudomonas putida* KT2440 wild type (*wt*) and mutants (*relA*/*spoT* and *rpoN*) grown under optimal (N+) and nitrogen limiting conditions (N−).

Analysis of the expression profiles showed that nitrogen limitation caused large changes in expression patterns. On the heatmap based on all genes (Figure 4), analyzed transcriptomes are significantly divided into two groups. The first of them consists of transcriptomes of the wild type and *rpoN* mutant cultured in nitrogen limiting conditions. The second group was created by the remaining transcriptomes. Among them, the closest were the transcriptomes of the wild type and *rpoN* mutant cultivated in optimal conditions. The first group was characterized by low expression level of all examined genes. Interestingly in the transcriptome of the *relA*/*spoT* mutant, some genes were downregulated whereas others were upregulated.

**Figure 5:**
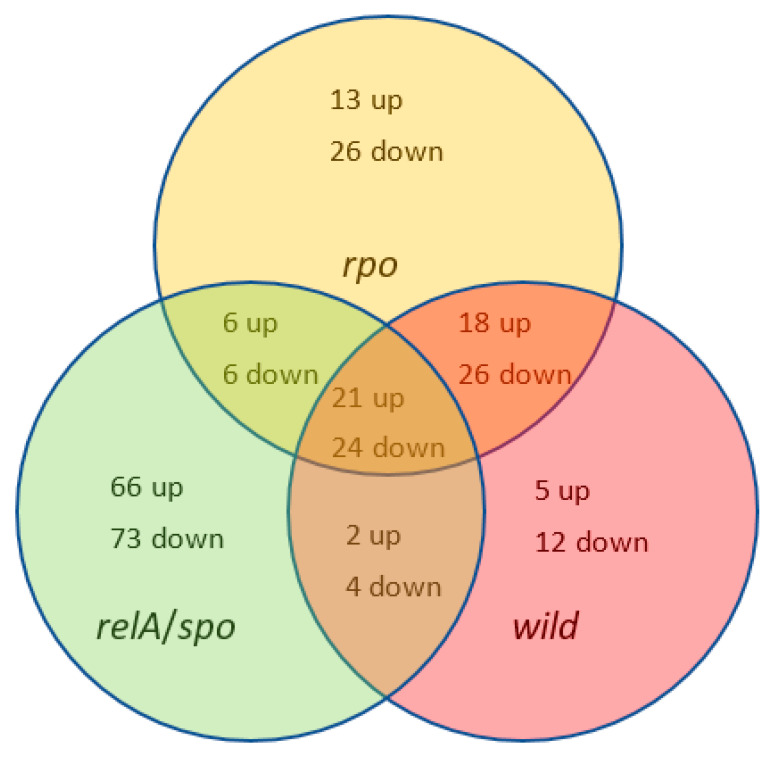
Venn diagram of genes that were differentially regulated under nitrogen limitation during growth of *Pseudomonas putida* KT2440 wild type (*wt*) and mutants (*relA*/*spoT* and *rpoN*).

To show the numbers of genes differing significantly between strains under nitrogen limitation conditions, a Venn diagram was created (Figure 5). The highest numbers of downregulated and upregulated genes were noticed in the *relA*/*spoT* mutant (73 and 66, respectively), whereas the lowest values were for the wild type (12 and 5, respectively). The most similar strains in terms of the number of genes showing any statistically significant alteration in expression were the wild strain and the *rpoN* mutant. The numbers of common downregulated and upregulated genes were 26 and 18, respectively. These values in the remaining two pairs were much lower. The numbers of common downregulated and upregulated genes for all pairs were 24 and 21, respectively.

**Figure 6:**
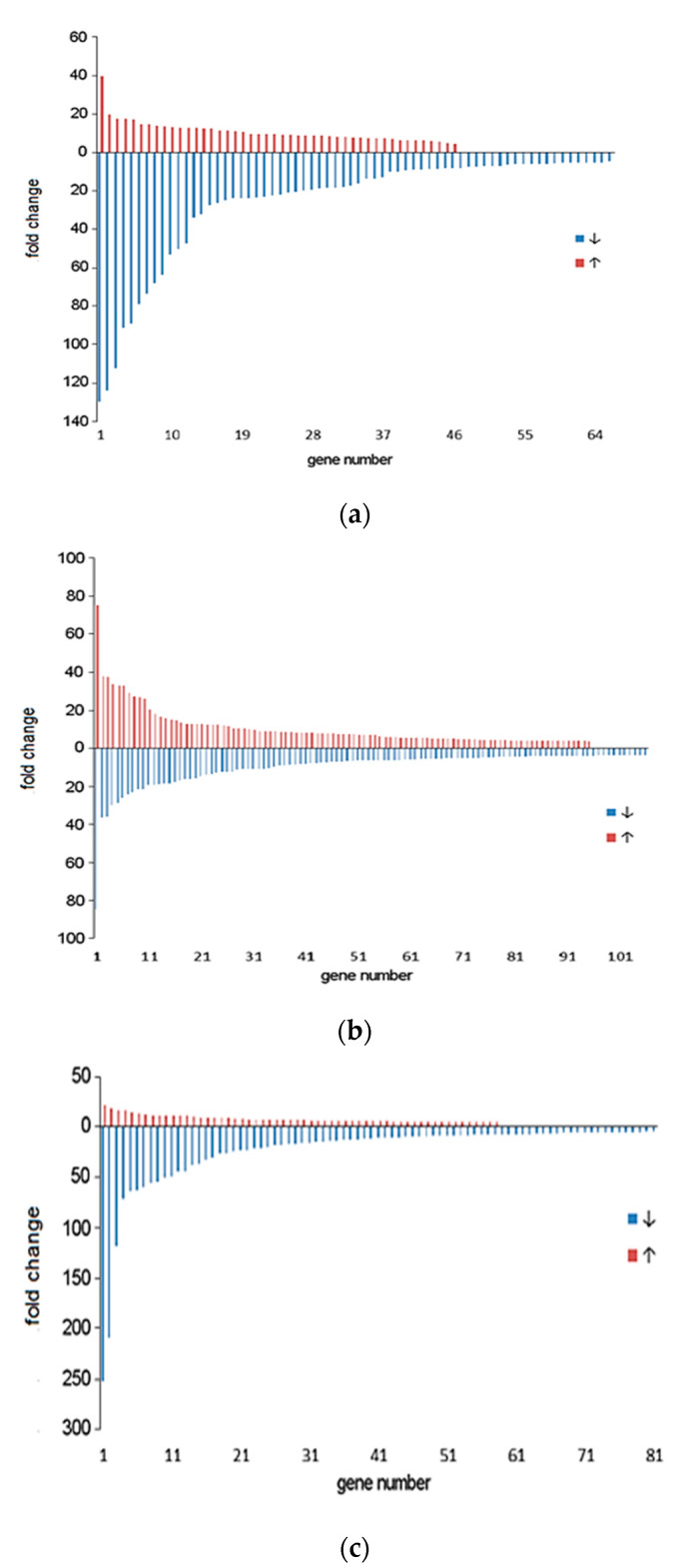
Distribution of fold changes in gene expression resulting from nitrogen limitation during cultivation of *Pseudomonas putida* KT2440 wild type (**a**) and *relA/spoT (***b***)* and *rpoN* mutants (**c**) (blue, downregulated; red, upregulated). The graph shows genes with statistically significant differences in expression.

The directions of gene transcription changes resulting from nitrogen limitation are shown in Figure 6. The lowest number of genes showing statistically significant expression changes was noticed in wild type. The number of genes showing upregulation (46 genes) was lower than the number of genes showing downregulation (66 genes). Additionally, the fold change of downregulated genes was much higher than that of upregulated genes. In *relA*/*spoT* mutant the numbers of genes showing upregulation and downregulation were similar (95 and 106, respectively). The values of fold changes were not higher than 40. The directions of transcription changes in *rpoN* mutant were similar to those of wild type. The number of genes showing upregulation (58 genes) was lower than the number of genes showing downregulation (81 genes). Similarly, to wild type the fold change of downregulation was much higher than upregulation.

The genes showing statistically significant downregulation in response to nitrogen limitation in all analyzed strains are listed in Table 1. Some of them were responsible for oxidative phosphorylation and code both for cytochrome bo terminal oxidase (cyoC and cyoD) and for cbb3-type cytochrome c oxidase (ccoO-I, ccoQ-I, ccoP-I). Additionally, this group comprises some genes responsible for proteins answering to cell stresses (cluster PP_3230, PP_3232, PP_3234–PP_3238). In this group, among others, genes coding for HSP20 family heat shock protein family and universal stress protein family were present. Among those genes, creating cluster PP_3289–PP_3292, and PP_3294, were also those related to stress. Additionally, significantly downregulated were genes responsible for catechol 1,2-dioxygenase, muconate cycloisomerase 1, and some unknown genes.

The genes showing statistically significant upregulation in response to nitrogen stress in all analyzed strains are listed in Table 2. Among 24 upregulated genes were those responsible for the nitrogen metabolism pathway (NirB, NirD, CobA, and NasA), ABC transporters (PP_2260, PP_2261, PP_2262, PP_2263, UrtA, UrtB, UrtC, UrtD), and chemotaxis (PP_0779, PP_4888). Other genes code flagellar hook protein (FlgE), transglutaminase (PP_2686), and undetermined, hypothetical proteins (PP_2687, PP_2688, PP_3007, and PP_4331).

### 2.4. Validation of Illumina Sequence Data Using qRT-PCR

To confirm the data obtained by RNAseq, the expression quantities of the most important genes for mcl-PHAs synthesis (*phaC1*, *phaZ*, *phaC2*, *phaD*, *phaI*, *phaF* and *phaG*) were determined with real-time quantitative PCR (qPCR). The results of qPCR were in good agreement with the RNAseq data, indicating that the data from RNAseq were of high quality (Table S1). Some discrepancies were observed in fold change values for wild type (*phaF* and *phaI*) and *rpoN* mutant (*phaZ* and *phaF*).

## 3. Discussion

Our results confirm that nitrogen limitation has a positive effect on mcl-PHA biosynthesis in *P. putida* KT2440. This change in environmental conditions caused mcl-PHA synthesis to increase in both the wild type and the *rpoN* mutant. It had already been shown that mcl-PHA synthesis in *P. putida* KT2440 and the RpoN negative mutant can be increased by nitrogen limitation when these strains are cultivated on gluconate (Hoffmann and Rehm, 2004). Our study, however, presents the novel finding that the *relA*/*spoT* mutant does not react to nitrogen depletion when cultivated on gluconate. In contrast, when the *relA*/*spoT* mutant is cultivated on oleic acid, nitrogen depletion does cause this mutant to increase mcl-PHA accumulation [21]. In our present study, when nitrogen was present in excess, all strains accumulated similar amounts of mcl-PHA (Figure 1). This suggests that the stringent response is not directly related to mcl-PHA synthesis but could be directly involved in fatty acid de novo biosynthesis. This could explain why the *relA*/*spoT* mutant did not synthesize mcl-PHAs when cultivated on gluconate.

RT-qPCR analysis of individual genes indicated that all analyzed genes were upregulated in the *P. putida* KT2440 *relA*/*spoT* mutant compared to the other strains. Interestingly, although expression levels of *phaC1*, *phaC2*, *phaI*, *phaF*, and *phaG* in this strain were higher in nitrogen limiting conditions than when nitrogen was present in excess, increased expression of these genes did not correspond to greater accumulation of mcl-PHAs. The transcription of all genes constituting the *phaC1*/*phaZ*/*phaC2*/*phaD* and *phaI*/*F* operons was significantly higher in the *relA*/*spoT* mutant than in the wild type. This suggests that the stringent response negatively regulates these genes, either directly or indirectly. In particular, we found that expression levels of the *phaI* and *phaF* genes were markedly higher in the *relA*/*spoT* mutant, which was also observed by Mozejko-Ciesielska et al. (2017) [21], when these strains were grown on oleic acid.

Our results do not corroborate a number of hypotheses or suggestions regarding regulation of mcl-PHA synthesis. First, we did not observe a positive correlation between transcription of *phaD* and that of *phaC1* genes. A similar lack of correlation was also observed when the *relA*/*spoT* mutant was grown on oleic acid [21]. Taken together, these studies indicate that the *phaD* gene is not necessarily an activator of the *phaC1* and *phaI* promoters, as was previously suggested [15,25]. Second, we found that phaF expression was similar in both the wild type and the *rpoN* mutant, which does not support the hypothesis that *phaF* is negatively regulated by RpoN [17].

In nitrogen limiting conditions in our study, expression of the gene coding for transacylase (*phaG*) was upregulated in all analyzed strains. However, whereas the increase in *phaG* expression in these conditions was similar in the wild type and the *relA*/*spoT* mutant, the increase in *phaG* expression in the *rpoN* mutant was smaller. This suggests that the stringent response regulates fatty acid de novo biosynthesis, but it does not influence the *phaG*. In addition, expression of *phaG* seems to be partially dependent on RpoN.

Under nitrogen limitation, gene expression in the *relA*/*spoT* mutant, which was deficient in the stringent response, differed from that in the wild type and the *rpoN* mutant. In the *relA*/*spoT* mutant, only some of the genes were downregulated under nitrogen limitation, whereas in the other two strains, the majority of the genes were downregulated in these conditions (Figure 4 and Figure 6). This suggests that the stringent response can globally regulate *P. putida* KT2440 metabolism.

In all three analyzed strains, 24 genes were significantly downregulated in nitrogen limiting conditions. Most of these genes are co-localized with genes possessing regulatory character (Figure S1), as indicated by inspection of the Pseudomonas.com database. Of these genes, the ones that code for cbb3-type cytochrome c oxidases (ccoO-I, ccoQ-I, ccoP-I) are putatively regulated by a DNA-binding transcriptional dual regulator (FnrA or ANR). Another set of these genes (PP_3230 and PP_3232–PP_3238), which includes genes coding for heat shock protein (HSP20 family, PP3234) and a universal stress protein (PP_3237), are believed to be regulated by a Crp/Fnr family transcriptional regulator (FnrB). Another group of these significantly differentially regulated genes, PP_3289–PP_3292, PP3294, PP_3713, PP_3715, and PP_3783, is believed to be regulated by another member of this family of regulators (FnrC). Interestingly, two of these genes (PP_3290, PP_3294) also code for stress proteins. Finally, two of these significantly differentially regulated genes code for cytochrome bo terminal oxidases (CyoC and CyoD) and are putatively controlled by the transcriptional regulator norR (Figure S1).

The function of cytochrome bo3 terminal oxidase (Cyo) is particularly important in *P. putida*, because it plays a dual role as both a terminal oxidase and as a component of a global regulation network. Levels of Cyo can change in response to changes in oxygen concentration and also depending on the carbon source that is being used [26,27]. Therefore, it seems that, when *P. putida* changes the composition of the electron transport chain to optimize energy generation, it can also influence the transcriptome profile of the cell through global control of gene expression, presumably to help with coordinating metabolism [28]. It has been reported that, in *P. putida* KT2440, transcription of one of the Cyo subunits (CyoA) is controlled by Anr [28]. In contrast, our analysis suggests that CyoC and CyoD are regulated by NorR (Figure S1). This protein binds to DNA upstream of the promoter site, and activates sigma^54^-dependent transcription of genes that encode nitric oxide detoxifying enzymes, which are produced in response to NO stress [29]. Expression of *cyoC* and *cyoD* was downregulated to a lesser extent in the *relA*/*spoT* mutant than in the wild type and the *rpoN* mutant.

Our results indicate that, under nitrogen limitation, the *fnrA* (ANR) and *fnrB* genes were not significantly induced or repressed in any of the studied strains (Table 1), although other genes putatively under the control of these genes were downregulated. Fumarate-nitrate reduction regulator (FNR) proteins are considered a major subgroup of the cyclic-AMP receptor protein family of bacterial transcription regulators. The major function of FNR proteins is to reprogram gene expression to coordinate the switch from aerobic to anaerobic metabolism when facultative anaerobes are starved of O_2_ [30]. The regulatory mechanisms of *fnrA* (ANR) have been studied in *Pseudomonas putida* by Ugidos et al. [28]. They discovered that, during exponential growth in a highly aerated complete medium, ANR activated expression of the cbb3-1 terminal oxidase, but had little effect on expression of cbb3-2 terminal oxidases. In our study, the *fnrC* gene exhibited strong, statistically significant downregulation in nitrogen limiting conditions in all studied strains, although, in the *relA*/*spoT* mutant, this change was much smaller than in the other strains. This suggests that the *fnrC* gene is controlled by the stringent response.

In all strains, if a gene was significantly downregulated, its putative regulator was usually not significantly downregulated. This observation suggests that other factors could influence the regulation of these genes. Additionally, some genes that are putatively controlled by the ANR regulon were downregulated to a smaller extent in the *relA*/*spoT* mutant than in the other strains. These genes code for three subunits of cbb3-type cytochrome c oxidase (ccoOQP-I) and HSP20 family heat shock protein (PP_3234). In contrast, the transcriptional regulator PyrR (PP_3238) was downregulated to a greater extent in the *relA*/*spoT* mutant than in the remaining strains. All these observations indicate that the ANR regulon is regulated by the stringent response in *P. putida* KT2440. Recently, it has become clear that the ANR regulon controls many more functions than was previously thought. In particular, it regulates many genes related to stress response and adaptability, as reported by Tribelli et al. [31]. The role of ANR in PHAs synthesis was also elucidated by Mohanan et al. (2019) in *P. chlororaphis* PA23 [32]. Interestingly, in our study there was an apparent association between the fold change of ccoOQP-I (Table 1) and the PHAs concentrations in cells under nitrogen limitation (Figure 1). In nitrogen limiting conditions, the lower the number of ccoOQP-I gene transcripts, the higher the mcl-PHAs concentration. This enzyme is a member of the heme-copper oxidase superfamily, and it plays a primary role in aerobic growth irrespective of oxygen concentration [33]. It was shown that, in *Ralstonia sphaeroides*, cbb3-type cytochrome c oxidase has a repressive role in the PrrBA-dependent expression of the photosynthesis genes [34,35]. Therefore, it is likely that ccoOQP-I also represses one or more genes involved in PHA synthesis. Otherwise, ccoOQP-I genes and genes involved in mcl-PHA synthesis are regulated in the same manner.

In nitrogen limiting conditions, only 21 genes were significantly upregulated in all three studied strains. Some of these genes are co-localized with genes with regulatory functions (Figure S2), according to the Pseudomonas.com database. Two genes coding for uroporphyrinogen-III C-methyltransferase (*cobA*) and nitrate transporter (*nasA*) are putatively regulated by *nasT* (PP_3093). In *P. aeruginosa*, the activity of NasT is negatively controlled by the nitrate sensitive regulator, NasS [36]. Therefore, it is likely that these genes are upregulated under nitrogen limitation. Another gene that could play a regulatory role is sigma^54^-dependent transcriptional regulator (PP_2259). This gene is responsible for regulation of sugar ABC transporters (PP_2260–PP_2263). Sigma^54^-dependent transcriptional regulator is dependent on RpoN. Therefore, in *rpoN* mutant cells, the expression of this gene is lower than in the wild type, as it also is in the *relA*/*spoT* mutant because RpoN is dependent on the presence of ppGpp [37]. Cells of some bacterial species respond to nitrogen starvation by activating the nitrogen regulation (Ntr) stress response [38]. In *P. putida*, PII protein controls the transcription of many nitrogen-dependent genes by regulating the kinase and phosphatase activities of NtrB, the sensor of the global two-component regulatory NtrB/NtrC system, thereby regulating the phosphorylation state of the transcriptional activator NtrC [39]. Genes arranged in two operons, from PP_2685 to PP_2688 and from PP_4841 to PP_4844, are likely to be controlled by NtrB/NtrC (Table 2). It has previously been shown by [39] that expression of the genes comprising the open reading frame from PP_2685 to PP_2688 is induced under nitrogen limitation. According to their investigation, PP_2685 is conserved in other groups of bacteria and shows similarity to the beta subunit of the 20S proteasome, which is an enzymatic complex for nonlysosomal protein degradation in both the cytosol and the nucleus [40]. It is probable that these genes are upregulated in nitrogen limiting conditions, and that they regulate other genes cooperating in proteolysis. Similarly, Poblete-Castro et al. (2012) has shown that the PP_2685 gene was upregulated in response to nitrogen limitation [10]. It was also shown by Hervás et al. (2007) that genes coding for urea ABC transporters were induced by NtrC under nitrogen starvation [41]. The regulation of the remaining eight genes, among which *nirB* and *nirD* were identified, was not clear.

We identified three putative gene regulators displaying statistically significant upregulation in nitrogen limiting conditions. These regulators were NasT (PP_2093), sigma^54^-dependent transcriptional regulator (PP_2259), and the two-component NtrB/NtrC regulator coded by the *glnL* gene (PP_5047) and the *glnG* gene (PP_5048), respectively. Of these genes, *nasT* and *glnL*/*glnG* exhibited higher expression in the *relA*/*spoT* mutant than in the other strains. In this mutant, increased expression of *nasT* did not influence the transcription of genes that are nasT-regulated. The expression levels of genes that are putatively regulated by NtrB/NtrC were higher in the *relA*/*spoT* mutant than in the wild type and *rpoN* mutant. These genes code for transglutaminase domain-containing protein, and UrtA, UrtB, UrtC, and UrtD, which are ABC transporters. This observation suggests that these genes are negatively regulated by the stringent response. The increased expression of *glnL* and *glnG* genes in the *relA*/*spoT* mutant could be surprising in the light of the results of Brown et al. (2014) [38]. They showed that transcription of *relA* is activated by NtrC during nitrogen starvation in *Escherichia coli,* linking in this way two major stress responses. Here, we showed that the stringent response could negatively regulate the NtrB/NtrC dual regulator. It is likely that, in *P. putida* KT2440, this mechanism differs from that in *E. coli*, as otherwise, two-way regulation (feedback) between these two stress responses would exist. In this scenario, *relA* could repress the activity of the NtrB/NtrC dual regulator.

## 4. Materials and Methods

### 4.1. Bacterial Strain and Growth Conditions

Cells of Pseudomonas putida KT2440 (ATCC^®^ 47054TM), *P. putida* KT2440 rpoN mutant [42], and *P. putida* KT2440 *relA*/*spoT* [43] mutant were taken from a deep-frozen stock and grown overnight in Luria Bertani broth (1% *w/v* tryptone, 0.5% *w/v* yeast extract, 1% NaCl) with shaking at 30 °C with 200 rpm for 24 h before inoculation. All studied strains were cultivated under nitrogen-limiting and non-limiting conditions. For all cultivations, the nitrogen-limited medium contained the following components per liter: 2 g Na_2_HPO_4_·12H_2_O, 14.9 g KCl, 46.72 g NaCl, 14.5 g Tris, 2.05 g MgCl_2_, 3.53 g Na_2_SO_4_, 1 g (NH_4_)SO_4_, 1 g MgSO_4_·7H_2_O, and 2.5 mL of trace element solution. In the non-limited experiments, the level of (NH_4_)SO_4_ was adjusted to 10 g/L. Each liter of trace element solution contained: 20 g FeCl_3_·6H_2_O, 10 g CaCl_2_·H_2_O, 0.03 g CuSO_4_·5H_2_O, 0.05 g MnCl_2_·4H_2_O, 0.1 g ZnSO_4_·7H_2_O dissolved in 0.5 N HCl. All cultures were supplemented with sodium gluconate (10 g/L) as the only carbon source in the production media. The 250 mL Erlenmeyer flasks containing 100 mL of a mineral medium were incubated for 48 h at 30 °C in a rotary shaker at 200 rpm.

### 4.2. Analytical Methods

The samples from shake flasks experiment were taken after 48 h of cultivation in order to measure ammonium concentration, cell dry mass and PHAs concentration. To measure cell dry mass (CDM), the cells in 100 mL culture broth were harvested by centrifugation at 11.200× *g* for 10 min, washed twice with distilled water. The collected cells were then weighed after lyophilization. The lyophilization process was performed by Lyovac GT2 System (SRK Systemtechnik GmbH) for 24 h. Ammonium concentration was measured spectrophotometrically using the Hach Lange DR 2800 spectrophotometer (Hach Lange, Düsseldorf DE) and the LCK303 kit for ammonium and LCK380 kit for TOC according to the manufacturer’s instructions.

Mcl-PHAs were extracted from lyophilized cells using the chloroform/methanol procedure for quantitative and qualitative analysis of biopolymers. The monomeric composition of the purified mcl-PHAs was determined using a methanolysis protocol as described previously [44]. The concentrations of methyl esters were estimated by a gas chromatography (GC) equipped with a capillary column Varian VF-5 ms with a film thickness of 0.25 μm (Varian, Lake Forest, USA). Pure standards of methyl 3-hydroxyhexanoate, 3-octanoate, 3-nonanoate, 3-decanoate, 3-undecanoate, 3-dodecanoate, 3-tetradecanoate, and 3-hexadecanoate (Larodan Fine Chemicals, Sweden) were used to generate calibration curves for the methanolysis assay. All samples were analyzed in triplicates. Student t-test was used to find statistically significant differences between biomass and PHAs concentration.

### 4.3. RNA Isolation

One aliquot of 1 mL from each of cultures were collected and centrifuged at 4000× *g* to pellet the cells and then transferred to a tube containing RNALater solution (Sigma). Total RNA extraction was performed using a commercial RNA extraction kit (A&A Biotechnology) according to the protocol described by Ciesielski et al. (2008) [45]. Isolated RNA samples were treated with the On-Column DNase I Digest Set (Sigma) to remove traces of DNA. Each time the absence of contaminating DNA was proven by PCR reaction. The RNA quantity, quality was checked using capillary electrophoresis (Agilent 2100 Bioanalyzer, CA, USA). The RNA integrity number (RIN) of every RNA sample used for sequencing was more than 8.0.

### 4.4. Reverse Transcription PCR Analysis

Reverse transcription was performed using a SuperScript ViloTM cDNA Synthesis Kit (Invitrogen) according to the manufacturer’s instructions. The cDNA reaction for each sample contained 1 μg of total RNA. Samples, without reverse transcriptase (RT) were used as a negative control. The synthesized first strand cDNA was suspended in sterile water and stored at −20 °C. Real-time PCR reaction was performed using SYBR Green technology in an ABI 7500 real-time PCR system (Applied Biosystems, USA) in MicroAmpTM Optical 96-well reaction plates (Applied Biosystems, USA). The primer pairs used for real-time amplification are given in Table S1. The reactions were run using the thermal cycling parameters as follows: 95 °C for 3 min; then 40 cycles of 95 °C for 15 s, and 60 °C for 1 min. After performing a run, a final standard melting curve stage was included. In each run, negative controls (no cDNA) for each primer set were included. For quantification of the fluorescence values, a calibration curve was made using dilution series from 5 × 10^−7^ to 5 ng of *P. putida* KT2440 genomic DNA sample. Normalized expression levels of the examined transcripts were estimated relative to the *16S rRNA* gene, as its expression is known to remain relatively constant throughout growth phase of *P. putida*. Then, the concentration of *P. putida* KT2440 DNA was converted to a genome equivalent for calculation of copy numbers in the real-time PCR assays [46]. For the convenience, the genome size of *P. putida* KT2440 (6.18 × 106 bp) available at NCBI (National Center for Biotechnology Information) was used to estimate the mean mass of the *P. putida* KT2440 genome accordingly to the equation:

*m* = (*n* × *mw*)/AN

where *n* is the genome size in base pairs, *mw* is the average molecular weight per base pairs (660 g mol^−1^), and AN is Avogadro’s constant (6023 × 1023 molecules mol^−1^).

### 4.5. Library Construction, Illumina Sequencing and Data Analysis

RNAseq template libraries were constructed with 1 μg of the enriched mRNA samples using Truseq RNA Sample Preparation Kit (Illumina, CA, USA) according to the manufacturer’s instructions. Deep sequencing was performed by Illumina HiSeq 2500 according to the manufacturer′s description with a read length of 1 × 50 nucleotides. Sequence reads were pre-processed to trim low-quality reads and filter reads shorter than 20 bp using FASTX Tool Kit. Genome sequences and annotation data of *P. putida* KT2440 were downloaded from NCBI. Reads that mapped to non-coding RNA sequences and reads that did not map to unique positions were excluded from further analysis. Remaining reads were mapped to *P. putida* KT2440 genome using Bowtie with the default parameters. The reads per gene values of all genes were calculated from the SAM output files. Testing for differential expression was performed with DESeq and R software package that uses a statistical model based on the negative binomial distribution [47]. Statistical analysis was performed, and genes with a false discovery rate (FDR) *p*-value correction < 0.01 were determined as differentially regulated genes.

## 5. Conclusions

Our study indicates that the stringent response influences mcl-PHA accumulation in *P. putida* KT2440 cultivated on sodium gluconate in nitrogen limiting conditions. Under nitrogen starvation, mcl-PHA accumulation is significantly lower in the *relA*/*spoT* mutant than in the wild type or *rpoN* mutant. Thus, accumulation of mcl-PHAs in *P. putida* KT2440 is not dependent on the RpoN transcription factor. *Quantitative PCR analysis indicated that* the *phaD* gene regulates the transcription of the *phaI* and *phaF* genes but not that of the *phaC1* gene. Additionally, we showed *that* the stringent response negatively regulates *phaI* and *phaF* expression. Expression of *phaG*, coding for transacylase, an enzyme that joins the pathways of *de novo* fatty acid and mcl-PHA synthesis, is induced by nitrogen limitation. Transcriptomic analysis revealed that the ANR regulon drives the reaction of *P. putida* to nitrogen stress mainly via the *fnrC* gene. We showed that this gene is downregulated by nitrogen limitation, except in the *relA*/*spoT* mutant. Thus, we provide evidence that transcription of *fnrC* is repressed by the stringent response under nitrogen starvation. We observed a strong negative association between cbb3-type cytochrome c oxidase gene (*ccoOQP-I)* expression and mcl-PHAs concentration. This could suggest that *ccoOQP-I* represses one or more of the genes directly involved in mcl-PHA synthesis. In addition, nitrogen limitation caused activation of the *nasT* regulator, which was particularly evident in the *relA*/*spoT* mutant. That indicates that this gene is also negatively regulated by the stringent response. Although it was shown in *E. coli* that the NtrB/NtrC dual regulator can influence the stringent response by *relA* activation, in *P. putida* KT2440, the stringent response may suppress expression of this regulator. Although our results do not entirely explain the network involved in the regulation of mcl-PHA synthesis, they do indicate possible directions for further investigation. In particular, the roles of the *fnrC*, *nasT*, and cbb3-type cytochrome c oxidase genes in response to nitrogen limitation, and their potential roles in mcl-PHA synthesis should be studied in more detail.

## Figures and Tables

**Table 1:** List of genes showing statistically significant downregulation detected in all analyzed strains as a result of nitrogen limitation. The values of fold change are given below strain names. *wt*—*P. putida* KT2440, *relA*/*spoT*—*P. putida* KT2440 *relA*/*spoT* mutant, *rpoN*—*P. putida* KT2440 *rpoN* mutant. The putative regulators of genes showing statistically significant differences in expression are given in grey fields. nd—not determined.

Gene ID	Gene Symbol	Protein	*wt*	*relA/spoT*	*rpoN*	*Putative Regulator*
**PP_4265**	**fnrA (Anr)**	**transcriptional regulator Anr**	**2.63**	**1.00**	**2.72**	
PP_4251	ccoO-I	cbb3-type cytochrome c oxidase subunit	123.89	16.95	252.3	fnrA (Anr)
PP_4252	ccoQ-I	cbb3-type cytochrome c oxidase subunit	91.18	18.42	209.35	fnrA (Anr)
PP_4253	ccoP-I	cbb3-type cytochrome c oxidase subunit	73.31	36.09	118.18	fnrA (Anr)
**PP_3233**	**fnrB**	**Crp/Fnr family transcriptional regulator**	**0.70**	**1.45**	**0.50**	
PP_3230	nd	phosphoribosyl transferase domain-containing protein	6.09	5.84	4.73	fnrB
PP_3232	nd	acetyltransferase	129.77	84.94	64.07	fnrB
PP_3234	nd	HSP20 family heat shock protein	112.06	19.24	70.71	fnrB
PP_3235	nd	hypothetical protein	18.07	6.67	10.98	fnrB
PP_3236	nd	lipoprotein OprI	23.43	16.39	16.47	fnrB
PP_3237	nd	universal stress protein family	20.45	17.67	16.73	fnrB
PP_3238	nd	transcriptional regulator PyrR	8.89	21.53	5.78	fnrB
**PP_3287**	**fnrC**	**Crp/Fnr family transcriptional regulator**	**42.46**	**1.21**	**36.46**	
PP_3289	nd	acetyltransferase	33.95	29.93	24.14	fnrC
PP_3290	nd	universal stress protein A family	23.31	11.31	14.06	fnrC
PP_3291	nd	metallo-beta-lactamase protein family	18.49	10.63	10.38	fnrC
PP_3292	nd	membrane protein	12.82	12.61	6.95	fnrC
PP_3294	catA-I	universal stress protein family	13.43	14.68	10.88	fnrC
PP_3713	catB	catechol 1,2-dioxygenase	19.44	4.22	5.62	nd
PP_3715	syrB	muconate cycloisomerase 1	32.00	19.07	4.44	nd
PP_3783	nd	syringomycin biosynthesis protein 2	8.42	5.00	5.81	nd
**PP_0807**	**norR**	**DNA-binding transcriptional regulator**	**0.49**	**0.87**	**0.65**	
PP_0814	cyoC	cyt. bo terminal oxidase subun III	26.22	7.43	15.99	norR
PP_0815	cyoD	cyt. bo terminal oxidase subunit IV	47.17	9.02	37.9	norR
PP_0989	gcvH-1	glycine cleavage system protein H	21.89	26.41	17.42	nd
PP_2745	nd	universal stress protein family	78.71	28.68	26.75	nd
PP_4837	nd	hypothetical protein	6.24	6.21	7.98	nd
PP_5735	nd	hypothetical protein	63.74	12.6	55.31	nd

**Table 2:** List of genes showing statistically significant upregulation detected in all analyzed strains as a result of nitrogen limitation. The values of fold change are given below strains names. *wt*—*P. putida* KT2440, *relA/spoT*—*P. putida* KT2440 *relA/spot* mutant, *rpoN*—*P. putida* KT2440 *rpoN* mutant. The putative regulators of genes showing statistically significant differences in expression are given in grey fields. nd—not determined, *—difference statistically not significant.

Gene ID	Gene Symbol	Protein	*wt*	*relA/spoT*	*rpoN*	Putative Regulator
**PP_2093**	***nasT***	**transcriptional regulator**	**7.25↑**	**27.66↑**	4.00↑*	
PP_2090	*cobA*	uroporphyrinogen-III C-methyltransferase	7.37	12.55	7.69	nasT
PP_2092	*nasA*	nitrate transporter	14.64	12.04	12.43	nasT
**PP_2259**	**nd**	**Sigma 54 dependent transcriptional regulator**	**2.00↓** *	1.75↑*	1.54↑*	
PP_2260	nd	glycerol-phosphate ABC transporter ATP-binding protein	17.36	4.18	6.74	PP_2259
PP_2261	nd	sugar ABC transporter ATP-binding protein	39.87	7.93	8.01	PP_2259
PP_2262	nd	sugar ABC transporter permease	11.31	6.05	6.29	PP_2259
PP_2263	nd	sugar ABC transporter permease	9.75	4.34	7.6	PP_2259
**PP_5047**	***glnL***	**ntrB**	1.00*	5.82↑*	**1.16↓** *	
**PP_5048**	***glnG***	**ntrC**	1.00*	4.84↑*	**1.23↓** *	
PP_2685	nd	hypothetical protein	1.12	4.81	1.45	ntrB/ntrC
PP_2686	nd	transglutaminase domain-containing protein	12.61	37.31	14.16	ntrB/ntrC
PP_2687	nd	hypothetical protein	11.04	16.57	16.02	ntrB/ntrC
PP_2688	nd	hypothetical protein	14.66	32.8	19.61	ntrB/ntrC
PP_4841	*urtA*	urea ABC transporter substrate-binding protein	12.7	75.03	11.12	ntrB/ntrC
PP_4842	*urtB*	urea ABC transporter permease	9.88	32.92	6.89	ntrB/ntrC
PP_4843	*urtC*	urea ABC transporter permease	14.23	26.17	9.35	ntrB/ntrC
PP_4844	*urtD*	ABC transporter ATP-binding protein	20.00	37.63	16.71	ntrB/ntrC
PP_0779	nd	methyl-accepting chemotaxis transducer/sensory box protein	12.6	5.46	11.26	nd
PP_1705	*nirB*	nitrite reductase large subunit	13.37	7.48	5.21	nd
PP_1706	*nirD*	nitrite reductase	17.03	10.49	6.57	nd
PP_3007	nd	hypothetical protein	7.68	5.13	7.95	nd
PP_4331	nd	hypothetical protein	6.33	3.84	6.71	nd
PP_4387	nd	hypothetical protein	13.22	3.53	11.64	nd
PP_4631	nd	hypothetical protein	7.97	8.95	7.12	nd
PP_4888	nd	methyl-accepting chemotaxis transducer	6.99	27.06	11.06	nd

## Data Availability

The data presented in this study are openly available in the NCBI (National Center for Biotechnology Information) Sequence Read Archive (SRA) under accession number SRP131442.

## Supplementary Materials

Supplementary materials can be found at https://www.mdpi.com/1422-0067/22/1/152/s1. Figure S1: Co-localization of genes showing statistically significant downregulation detected in all analyzed strains as a result of nitrogen limitation (A—*fnrA*, A—*fnrB*, C—*fnrC*, D—*norR*). Figure S2: Co-localization of genes showing statistically significant upregulation detected in all analyzed strains as a result of nitrogen limitation (A—PP_2093, B—PP_2259). Table S1. Validation of RNA-seq data by quantitative PCR using genes directly involved in mcl-PHA synthesis. *wt*—*P. putida* KT2440, *relA/spoT*—*P. putida* KT2440 *relA/spot* mutant, *rpoN*—*P. putida* KT2440 *rpoN* mutant.

## References

[1] Volova T.G., Zhila N.O., Shishatskaya E., Mironov P.V., Vasil’Ev A.D., Sukovatyi A.G., Sinskey A.J. (2013). The physicochemical properties of polyhydroxyalkanoates with different chemical structures. Polym. Sci. Ser. A.

[2] Kai D., Loh X. (2014). Polyhydroxyalkanoates: Chemical Modifications Toward Biomedical Applications. ACS Sustain. Chem. Eng..

[3] Anderson A.J., Dawes E.A. (1990). Occurrence, metabolism, metabolic role, and industrial uses of bacterial polyhydroxyalkanoates. Microbiol. Rev..

[4] Prieto A., Escapa I.F., Martínez V., Dinjaski N., Herencias C., De La Peña F., Tarazona N., Revelles O. (2016). A holistic view of polyhydroxyalkanoate metabolism in Pseudomonas putida. Environ. Microbiol..

[5] Madison L.L., Huisman G.W. (1999). Metabolic Engineering of Poly(3-Hydroxyalkanoates): From DNA to Plastic. Microbiol. Mol. Biol. Rev..

[6] Mozejko-Ciesielska J., Szacherska K., Marciniak P. (2019). Pseudomonas Species as Producers of Eco-friendly Polyhydroxyalkanoates. J. Polym. Environ..

[7] Luef K.P., Stelzer F., Wiesbrock F. (2015). Poly(hydroxy alkanoate)s in Medical Applications. Chem. Biochem. Eng. Q..

[8] Ankenbauer A., Schäfer R.A., Viegas S.C., Pobre V., Voß B., Arraiano C.M., Takors R. (2020). Pseudomonas putida KT2440 is naturally endowed to withstand industrial-scale stress conditions. Microb. Biotechnol..

[9] Follonier S., Escapa I.F., Fonseca P.M., Henes B., Panke S., Zinn M., Prieto M.C. (2013). New insights on the reorganization of gene transcription in Pseudomonas putida KT2440 at elevated pressure. Microb. Cell Factories.

[10] Poblete-Castro I., Escapa I.F., Jäger C., Puchalka J., Chi Lam C.M.C., Schomburg D., Prieto M.A., Martins Dos Santos V.A.P.M. (2012). The metabolic response of P. putida KT2442 producing high levels of polyhydroxyalkanoate under single- and multiple-nutrient-limited growth: Highlights from a multi-level omics approach. Microb. Cell Factories.

[11] Jimenez J.I., Minambres B., Garcia J.L., Díaz E. (2002). Genomic analysis of the aromatic catabolic pathways from Pseudomonas putida KT2440. Environ. Microbiol..

[12] Kim J., Oliveros J.C., Nikel P.I., De Lorenzo V., Silva-Rocha R. (2013). Transcriptomic fingerprinting of Pseudomonas putida under alternative physiological regimes. Environ. Microbiol. Rep..

[13] Beckers V., Poblete-Castro I., Tomasch J., Wittmann C. (2016). Integrated analysis of gene expression and metabolic fluxes in PHA-producing Pseudomonas putida grown on glycerol. Microb. Cell Factories.

[14] Prieto M.A., De Eugenio L.I., Galán B., Luengo J.M., Witholt B. (2007). Synthesis and Degradation of Polyhydroxyalkanoates. Pseudomonas.

[15] De Eugenio L.I., Escapa I.F., Morales V., Dinjaski N., Galán B., García J.L., Prieto M.A. (2010). The turnover of medium-chain-length polyhydroxyalkanoates inPseudomonas putidaKT2442 and the fundamental role of PhaZ depolymerase for the metabolic balance. Environ. Microbiol..

[16] Hoffmann N., Amara A.A., Beermann B.B., Qi Q., Hinz H.-J., Rehm B.H.A. (2002). Biochemical Characterization of the Pseudomonas putida3-Hydroxyacyl ACP:CoA Transacylase, Which Diverts Intermediates of Fatty Acid de Novo Biosynthesis. J. Biol. Chem..

[17] Hoffmann N., Rehm B.H.A. (2005). Nitrogen-dependent regulation of medium-chain length polyhydroxyalkanoate biosynthesis genes in pseudomonads. Biotechnol. Lett..

[18] Ciesielski S., Mozejko J., Przybyłek G. (2010). The influence of nitrogen limitation on mcl-PHA synthesis by two newly isolated strains of Pseudomonas sp.. J. Ind. Microbiol. Biotechnol..

[19] Mozejko-Ciesielska J., Pokoj T., Ciesielski S. (2018). Transcriptome remodeling of Pseudomonas putida KT2440 during mcl-PHAs synthesis: Effect of different carbon sources and response to nitrogen stress. J. Ind. Microbiol. Biotechnol..

[20] Brigham C.J., Speth D.R., Rha C., Sinskey A.J. (2012). Whole-Genome Microarray and Gene Deletion Studies Reveal Regulation of the Polyhydroxyalkanoate Production Cycle by the Stringent Response in Ralstonia eutropha H16. Appl. Environ. Microbiol..

[21] Mozejko-Ciesielska J., Dabrowska D., Szalewska-Palasz A., Ciesielski S. (2017). Medium-chain-length polyhydroxyalkanoates synthesis by Pseudomonas putida KT2440 relA/spoT mutant: Bioprocess characterization and transcriptome analysis. AMB Express.

[22] Potrykus K., Cashel M. (2008). (p)ppGpp: Still Magical?. Annu. Rev. Microbiol..

[23] Belda E., Van Heck R.G.A., Lopez-Sanchez M.J., Cruveiller S., Barbe V., Fraser C., Klenk H.-P., Petersen J., Morgat A., Nikel P.I. (2016). The revisited genome ofPseudomonas putidaKT2440 enlightens its value as a robust metabolicchassis. Environ. Microbiol..

[24] Mortazavi A., Williams B.A., McCue K., Schaeffer L., Wold B. (2008). Mapping and quantifying mammalian transcriptomes by RNA-Seq. Nat. Methods.

[25] Klinke S., De Roo G., Witholt B., Kessler B. (2000). Role of phaD in Accumulation of Medium-Chain-Length Poly(3-Hydroxyalkanoates) in Pseudomonas oleovorans. Appl. Environ. Microbiol..

[26] Dinamarca M.A., Ruiz-Manzano A., Rojo F. (2002). Inactivation of Cytochrome o Ubiquinol Oxidase Relieves Catabolic Repression of the Pseudomonas putida GPo1 Alkane Degradation Pathway. J. Bacteriol..

[27] Dinamarca M.A., Aranda-Olmedo I., Puyet A., Rojo F. (2003). Expression of the Pseudomonas putida OCT Plasmid Alkane Degradation Pathway Is Modulated by Two Different Global Control Signals: Evidence from Continuous Cultures. J. Bacteriol..

[28] Ugidos A., Morales G., Rial E., Williams H.D., Rojo F. (2008). The coordinate regulation of multiple terminal oxidases by the Pseudomonas putida ANR global regulator. Environ. Microbiol..

[29] Tucker N.P., Ghosh T., Bush M., Zhang X., Dixon R. (2009). Essential roles of three enhancer sites in σ54-dependent transcription by the nitric oxide sensing regulatory protein NorR. Nucleic Acids Res..

[30] Ibrahim S.A., Crack J.C., Rolfe M.D., Borrero-De Acuña J.M.B.-D., Thomson A.J., Le Brun N.E., Schobert M., Stapleton M.R., Green J. (2015). ThreePseudomonas putidaFNR Family Proteins with Different Sensitivities to O2. J. Biol. Chem..

[31] Tribelli P.M., Luján A.M., Pardo A., Ibarra J.G., Fernández Do Porto D.F.D., Smania A., López N.I. (2019). Core regulon of the global anaerobic regulator Anr targets central metabolism functions in Pseudomonas species. Sci. Rep..

[32] Mohanan N., Gislason A., Sharma P.K., Ghergab A., Plouffe J., Levin D.B., De Kievit T. (2019). Quorum sensing and the anaerobic regulator (ANR) control polyhydroxyalkanoate (PHA) production in Pseudomonas chlororaphis PA23. FEMS Microbiol. Lett..

[33] Arai H. (2011). Regulation and Function of Versatile Aerobic and Anaerobic Respiratory Metabolism in Pseudomonas aeruginosa. Front. Microbiol..

[34] O’Gara J.P., Eraso J.M., Kaplan S. (1998). A Redox-Responsive Pathway for Aerobic Regulation of Photosynthesis Gene Expression in Rhodobacter sphaeroides 2.4.1. J. Bacteriol..

[35] Oh J.-I., Kaplan S. (1999). The *cbb_3_* Terminal Oxidase of *Rhodobacter sphaeroides* 2.4.1: Structural and Functional Implications for the Regulation of Spectral Complex Formation. Biochemistry.

[36] Romeo A., Sonnleitner E., Sorger-Domenigg T., Nakano M., Eisenhaber B., Bläsi U. (2012). Transcriptional regulation of nitrate assimilation in Pseudomonas aeruginosa occurs via transcriptional antitermination within the nirBD–PA1779–cobA operon. Microbiology.

[37] Szalewska-Palasz A., Johansson L.U.M., Bernardo L.M.D., Skärfstad E., Stec E., Brännström K., Shingler V. (2007). Properties of RNA Polymerase Bypass Mutants: Implications for the role of ppGpp and its co-factor DksA in controlling transcription dependent on σ54. J. Biol. Chem..

[38] Brown D.R., Barton G.J., Pan Z., Buck M., Wigneshweraraj S. (2014). Nitrogen stress response and stringent response are coupled in *Escherichia coli*. Nat. Commun..

[39] Hervás A.B., Canosa I., Little R., Dixon R., Santero E. (2009). NtrC-Dependent Regulatory Network for Nitrogen Assimilation in Pseudomonas putida. J. Bacteriol..

[40] Gille A., Goede A., Schlöetelburg C., Preissner R., Kloetzel P.-M., Göbel U.B., Frömmel C. (2003). A Comprehensive View on Proteasomal Sequences: Implications for the Evolution of the Proteasome. J. Mol. Biol..

[41] Hervás A.B., Canosa I., Santero E. (2007). Transcriptome Analysis of Pseudomonas putida in Response to Nitrogen Availability. J. Bacteriol..

[42] Köhler T., Harayama S., Ramos J.L., Timmis K.N. (1989). Involvement of Pseudomonas putida RpoN sigma factor in regulation of various metabolic functions. J. Bacteriol..

[43] Sze C.C., Bernardo L.M.D., Shingler V. (2002). Integration of global regulation of two aromatic-responsive sigma(54)-dependent systems: A common phenotype by different mechanisms. J. Bacteriol..

[44] Mozejko J., Przybyłek G., Ciesielski S. (2011). Waste rapeseed oil as a substrate for medium-chain-length polyhydroxyalkanoates production. Eur. J. Lipid Sci. Technol..

[45] Ciesielski S., Pokój T., Klimiuk E. (2008). Molecular insight into activated sludge producing polyhydroxyalkanoates under aerobic–anaerobic conditions. J. Ind. Microbiol. Biotechnol..

[46] Cottyn B., Baeyen S., Pauwelyn E., Verbaendert I., De Vos P., Bleyaert P., Höfte M., Maes M. (2010). Development of a real-time PCR assay for Pseudomonas cichorii, the causal agent of midrib rot in greenhouse-grown lettuce, and its detection in irrigating water. Plant Pathol..

[47] Anders S., Huber W., Nagalakshmi U., Wang Z., Waern K., Shou C., Raha D., Gerstein M., Snyder M., Mortazavi A. (2010). Differential expression analysis for sequence count data. Genome Biol..

