# Supplementary material for: Transcriptome Changes in Pseudomonas putida KT2440 during Medium-Chain-Length Polyhydroxyalkanoate Synthesis Induced by Nitrogen Limitation
Source: Int J Mol Sci. 2020 Dec 25;22(1):152. doi: 10.3390/ijms22010152 (PMC7801951; doi:10.3390/ijms22010152)
Supplement: Supplementary file 1 [file ijms-22-00152-s001.zip › Figure S2.docx]

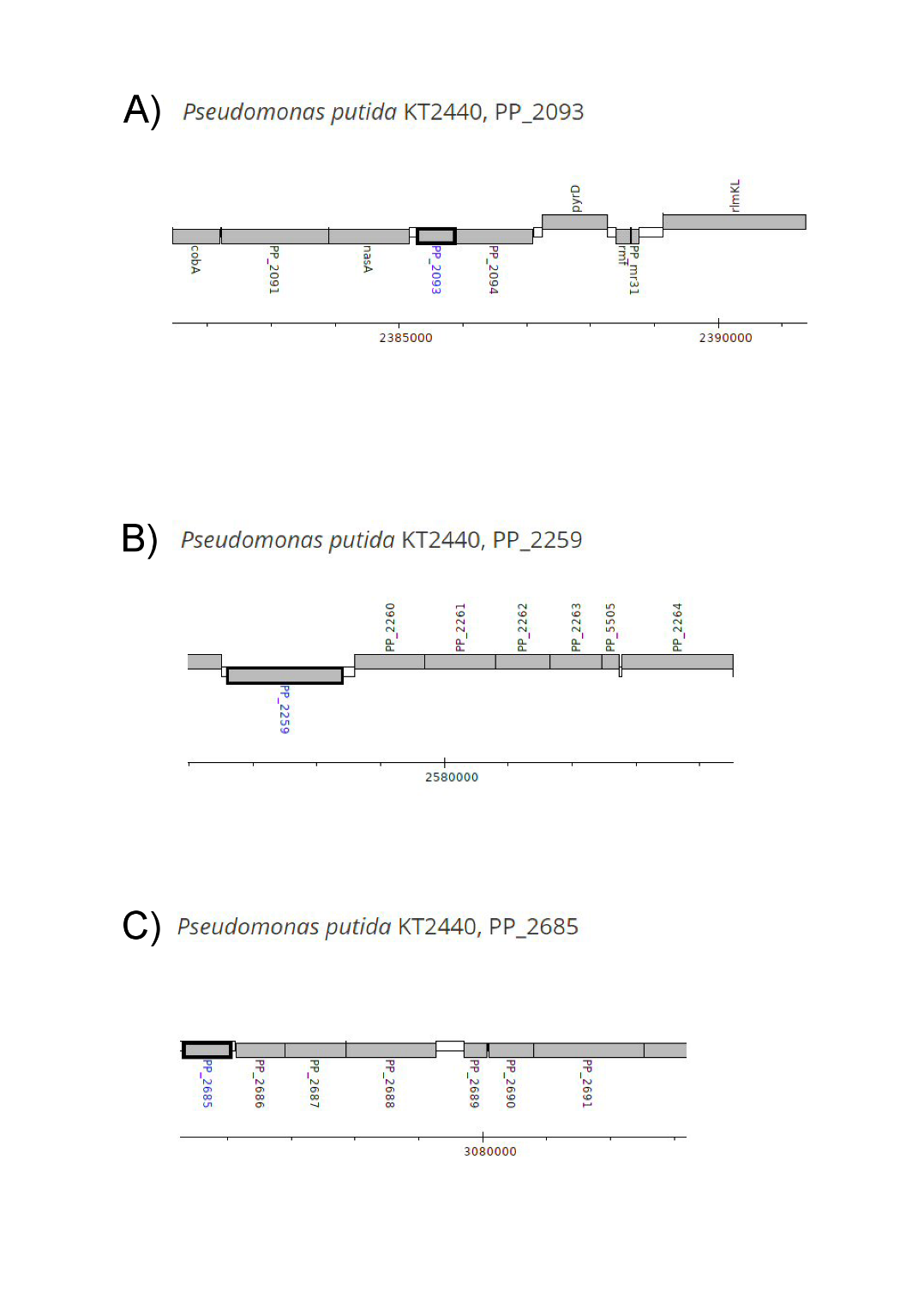


Figure S2. Co-localization of genes showing statistically significant upregulation detected in all analysed strains as the result of nitrogen limitation (A – PP_2093, B – PP_2259).
