# Supplementary material for: Transcriptome Changes in Pseudomonas putida KT2440 during Medium-Chain-Length Polyhydroxyalkanoate Synthesis Induced by Nitrogen Limitation
Source: Int J Mol Sci. 2020 Dec 25;22(1):152. doi: 10.3390/ijms22010152 (PMC7801951; doi:10.3390/ijms22010152)
Supplement: Supplementary file 1 [file ijms-22-00152-s001.zip › Table S1.docx]

Table S1. Validation of RNA-seq data by quantitative PCR using genes directly involved in mcl-PHA synthesis. *wt* – *P. putida* KT2440, *relA/spoT* – *P. putida* KT2440 *relA/spoT* mutant, *rpoN* – *P. putida* KT2440 *rpoN* mutant.

| **Gene / ID** | **strain** | **fold change** | |
| --- | --- | --- | --- |
|  |  | **qPCR** | **RNA-seq** |
| ***phaC1 / PP_5003*** | *wt* | 0.85 | 0.60 |
|  | *relA/spoT* | 1.23 | 1.10 |
|  | *rpoN* | 0.39 | 0.40 |
| ***phaZ / PP_5004*** | *wt* | 0.90 | 0.70 |
|  | *relA/spoT* | 0.95 | 0.88 |
|  | *rpoN* | 1.04 | 0.69 |
| ***phaC2 / PP_5005*** | *wt* | 0.97 | 0.97 |
|  | *relA/spoT* | 1.83 | 1.01 |
|  | *rpoN* | 1.47 | 0.79 |
| ***phaD / PP_5006*** | *wt* | 1.54 | 1.21 |
|  | *relA/spoT* | 0.90 | 0.76 |
|  | *rpoN* | 1.33 | 1.24 |
| ***phaF / PP_5007*** | *wt* | 1.12 | 0.82 |
|  | *relA/spoT* | 1.47 | 2.31 |
|  | *rpoN* | 1.01 | 0.59 |
| ***phaI / PP_5008*** | *wt* | 1.84 | 0.37 |
|  | *relA/spoT* | 1.66 | 1.89 |
|  | *rpoN* | 0.85 | 0.76 |
| ***phaG / PP_1408*** | *wt* | 4.16 | 2.94 |
|  | *relA/spoT* | 3.41 | 2.74 |
|  | *rpoN* | 1.74 | 1.83 |
